# Sudden Cardiac Death in Athletes: From the Basics to the Practical Work-Up

**DOI:** 10.3390/medicina57020168

**Published:** 2021-02-14

**Authors:** Adriano Nunes Kochi, Giulia Vettor, Maria Antonietta Dessanai, Francesca Pizzamiglio, Claudio Tondo

**Affiliations:** 1 Heart Rhythm Center, Department of Clinical Electrophysiology and Cardiac Pacing at Monzino Cardiology Center, IRCCS, 20138 Milan, Italy; adrianokochi@gmail.com (A.N.K.); giulia.vettor@cardiologicomonzino.it (G.V.); mariantonietta.dessanai@cardiologicomonzino.it (M.A.D.); francesca.pizzamiglio@cardiologicomonzino.it (F.P.); 2 Nossa Senhora da Conceição Hospital, 91350-200 Porto Alegre, Brazil; 3 Department of Biochemical, Surgical and Dentist Sciences, University of Milan, 20122 Milan, Italy

**Keywords:** sudden cardiac death, athletes, ventricular arrhythmias, hypertrophic cardiomyopathy, myocarditis, arrhythmogenic right ventricular cardiomyopathy, channelopathies, ICD implant

## Abstract

Sudden cardiac death in athletes is a relatively rare event, but due to the increasing number of individuals practicing high-performance sports, in absolute terms, it has become an important issue to be addressed. Since etiologies are many and the occurrence is rare, tracing the ideal preparticipation screening program is challenging. So far, as screening tools, a comprehensive clinical evaluation and a simple 12-lead electrocardiogram (ECG) seem to be the most cost-effective strategy. Recent technological advances came to significantly help as second-line investigation tools, especially the cardiac magnetic resonance, which allows for a more detailed ventricular evaluation, cardiac tissue characterization, and eliminates the poor acoustic window problem. This article aims to review all aspects related to sudden cardiac death in athletes, beginning with definitions and epidemiology, passing through etiology and clinical characteristics, then finishing with a discussion about the best ambulatory investigational approach.

## 1. Introduction

It is public knowledge that physical activity promotes health because once practiced properly, it brings benefits regardless of age, sex, or ethnicity. Despite this, rare, sudden cardiac death may occur in the exercise context. When it happens to famous athletes, it reaches the journalistic spotlight and tends to cause public clamor since they are public figures and symbols of health. Strategies trying to mitigate it have been developed by the local arrhythmia societies. However, the development and implementation of these guidelines are challenging due to many factors such as the non-unanimous athlete definition, the varying etiology of sudden cardiac death between different ages and the geographic areas, and the difficulty to develop studies in the field due to the low incidence.

## 2. Definitions

Sudden cardiac death (SCD) is defined by the 2015 Ventricular Arrhythmias and the Prevention of Sudden Cardiac Death European Society of Cardiology (ESC) Guidelines as an unexpected, non-traumatic death within 1 h of symptoms’ onset in a patient known to have a potentially fatal cardiac condition, or when autopsy finds a cardiac or vascular anomaly as the probable cause, or when no extracardiac causes are found in the post-mortem examination, and therefore an arrhythmic event is the likely cause of death [1].

The definition of an athlete, on the other hand, is not homogeneous between epidemiological studies evaluating cardiac events. Most often, an athlete is defined as a regular exercise practitioner (force or endurance) aiming for participation in competitions. The Bethesda conference, commonly used as a guideline to decide for exercise qualification, defines an athlete as: “*One who participates in an organized team or individual sport that requires regular competition against others as a central component, places a high premium on excellence and achievement, and requires some form of systematic (and usually intense) training*”. On the other hand, ESC defines the same as: “*Individuals of young and adult age, either amateur or professional, who are engaged in exercise training on a regular basis and participate in official sports competition. Official sports competition (local, regional, national, or international) is defined as an organized team or individual sports event that, placing a high premium on athletic excellence and achievement, is organized and scheduled in the agenda of a recognized Athletic Association*”. Despite being clear about the term athlete, both definitions ignore the growing existence of the exerciser: the person that does regular physical exercise, and at times very intense, but never participates in organized competitions. This individual is precisely the most frequently seen in cardiology medical visits and pre-sports training evaluation. Pragmatically, this individual should be evaluated in the same way a competing athlete would be. Although, some authors defend that they may have different cardiovascular risk profiles since an exerciser would be primarily motivated by health improvement, while an athlete would be mainly motivated to win the competition, which can lead to exaggerated training and/or substance abuse, despite the control that has been put in practice by many sports organizations [1,2].

## 3. Epidemiology

In a world where the leading cause of death is ischemic heart disease, exercise practice is a growing tendency. The New York Marathon had, in 2019, 53,627 finishers, an impressive number for a competition with such rigid criteria for participation. The self-reported evaluation of physical activity worldwide published in 2003 had already observed 31.4% of adults declaring to practice vigorous-intensity physical activity at least three times per week [3].

The real incidence of SCD in athletes is very controversial due to the heterogeneity of the available data. Between the studies, neither the athlete definition is unanimous (denominator), nor the SCD event (numerator). Some studies considered only the fatal events, while others also considered persons that were successfully resuscitated or revived.

A retrospective forensic study developed in Spain evaluated the epidemiological data of 645 SCD victims, 1 to 35 years old, from 2010 to 2017. In this cohort, 75 (11.6%) of the events were exercise-related. Myocardial disease was diagnosed in 33 patients, arrhythmogenic cardiomyopathy being the leading cause (37%), followed by hypertrophic cardiomyopathy (24%) and myocarditis (15%). Only five had a previous cardiomyopathy diagnosis, and 85% were practicing recreational sports [4].

In Italy, Corrado et al. evaluated a 26-year trend in SCD in athletes (12 to 35 years old), and observed an incidence of 1.9/100,000 person-years [5].

An epidemiological study in high school athletes from Minnesota state, USA, over 12 years found three SCD events, an incidence of 1:500,000 competitive sports participants. Over a 3-year high school career for a student-athlete, the estimated risk was 1:72,500 [6].

In Israel, a study led by Steinvil et al. searched for SCD in athletes in the two main newspapers from 1985 to 2009. Using data from the Israel Sports Authority as the denominator, the calculated incidence of SCD in this context was 2.6/100,000 athlete-years [7].

A study from Sweden evaluated all SCDs in 10- to 35-year-old individuals from 2000–2010, retrieving information from medical records, death certificates, and autopsy data. In the 10-year period, 514 SCDs events were observed, 62 (12%) being considered exercise-related. Of those individuals, 21 were considered athletes and 41 non-athletes. The leading causes were primary arrhythmic heart diseases, followed by hypertrophic cardiomyopathy. Unfortunately, in this study, no denominator was estimated, so it did not provide us an incidence [8].

A study run in the USA evaluated, from 2003 to 2013, a database from the National Collegiate Athletic Association. They observed a total of 79 SCDs, an incidence of 1:53,703 athletes-year. In this study, most commonly, the autopsy was negative for cardiac structural abnormalities, followed by coronary anomalies (11%) and hypertrophic cardiomyopathy (10%). A total of 40 patients had a previous ECG, and 67% of these ECGs had T wave inversion [9].

Evaluating adolescents specifically, a study from 1996 to 2016 screened 11,168 adolescent athletes registered in the English Football Association Cardiac Screening Program (mean age 16 years old). In this group, the SCD incidence was 6.8 per 100,000 person-years [10].

## 4. Risk Factors

A study from Sweden evaluated all SCD events in 10- to 35-year-old Swedish patients from 2000 to 2010. In this cohort of 514 patients, 12% were exercise-related. In the subgroup of exercise-related, 90% were men. The most frequent etiologies found in autopsy were: sudden arrhythmic death syndrome (24%), hypertrophic cardiomyopathy (16%), and arrhythmogenic cardiomyopathy (13%). The premortal diagnosis was known in 27%; 13% had cardiac symptoms, and 31% had a family history of SCD, heart disease, or familial hypercholesterolemia [8].

In the United States, the RACER Study Group evaluated runners from marathons and half-marathons in the U.S. from January 2000 to May 2010. The incidence of SCD was 0.54/100,000 participants. The incidence in marathons was 3.7 times higher compared to half-marathons and 5.6 times higher in men. From the 59 events, only 31 had medical information to be certain about the cause of death. Hypertrophic cardiomyopathy was not only the leading cause but also the predictor of non-survival of the event [11].

Maron et al. studied, over a 10-year period, the causes and clinical profile of 182 athletes registered in national college registries. In this cohort, cardiovascular deaths were 5-fold more common in African-American athletes than in white athletes (3.8 vs. 0.7/100,000 athlete participation-years; *p* < 0.01) [12].

A nested case-crossover study from Albert et al. evaluated the SCD risk during vigorous exercise and 30 min after. A total of 122 SCDs were observed in a cohort of 21,518 male physicians in 12 years of follow-up. Vigorous exercise elevated the risk of SCD by a factor of 14 to 45. In the same context, they observed that the habitus of practicing vigorous exercise was a mitigating factor to the risk of SCD [13].

Again, it is hard to be sure about risk factors for SCD in athletes, but looking at the evidence, despite somewhat heterogeneous, it points that male sex, African-American ethnicity, the presence of structural or electrical heart diseases, and vigorous exercise (especially in starters) are risk factors that should be taken into account. Table 1 summarizes the risk factors [14,15].

## 5. Specific Conditions

### Hypertrophic Cardiomyopathy

Hypertrophic cardiomyopathy (HCM) is the most common genetic cardiovascular disease, being found in up to 1 of every 500 individuals, with equal predominance in men and women. The autosomal dominant mutation causes disarray in the cardiac sarcomeres, the most frequently muted genes being the MYH7 and the MYBPC3. The phenotypic presentation of the disease is a hypertrophied left ventricle (LV), predominantly in the septum. The ESC 2014 guidelines consider a LV wall thickness of ≥15 mm as diagnostic criteria, not explained by loading conditions [16].

In a study by Lorenzini et al., in a median follow-up of 6.2 years, the incidence of all-cause mortality in HCM patients was 12.4%. Compared to the general population, the excess mortality was lowered after the year 2000, probably related to the widespread implantable cardiac defibrillator (ICD) implant [17].

HCM is historically known as the main cause of SCD in patients younger than 35 years old. However, it is important to remember that it may not be true for different regions/countries. In the U.S. National Registry of Sudden Death in Athletes, HCM was responsible for 36% of SCD cases [18]. On the other hand, in a very similar study in the U.K., definitive HCM was diagnosed in only 6% of the SCD cases; however, idiopathic left ventricular hypertrophy was present in another 16%, raising the question of whether it represents an initial form of HCM [19].

The last American College of Cardiology (ACC)/The American Heart Association (AHA) guidelines consider an indication for ICD implant for primary prevention: family history of SCD in first-degree relatives, LV wall thickness of ≥30 mm, and recent unexplained syncope. The ESC guidelines, on the other hand, use the calculated risk as a percentage. In the same context, ICD implantation has a IIb recommendation level when the 5-year risk is ≥4% and a IIa recommendation level if the risk is ≥6% [16,20].

Despite most of the ICD therapies for ventricular tachycardia (VT) and ventricular fibrillation (VF) happening not in the exercise context, both guidelines advise against participation in high-intensity sports, even for those with ICD already implanted.

## 6. Arrhythmogenic Cardiomyopathy

**Figure 1:**
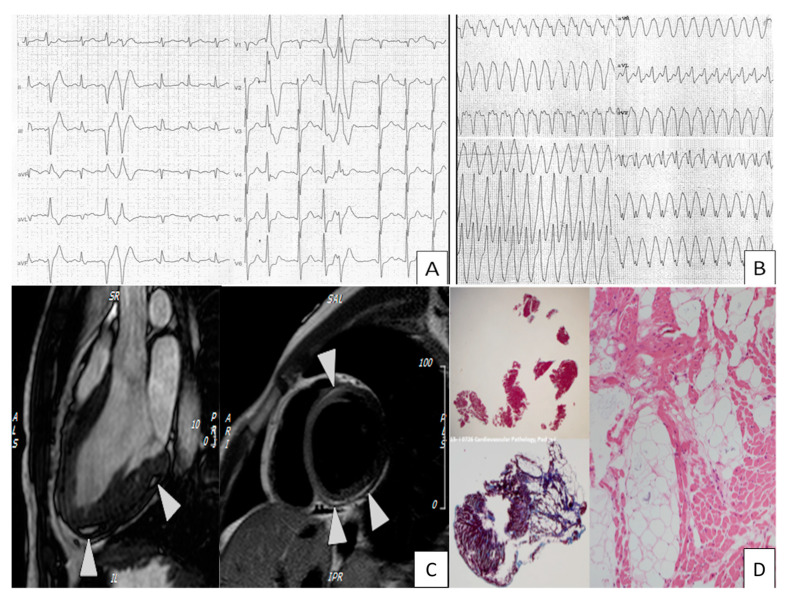
Findings in a diagnostic work-up of arrhythmogenic cardiomyopathy. (**A**) Frequent and coupled premature ventricular beats during exercise testing. (**B**) Sustained ventricular tachycardia. (**C**) CMR revealing adipose infiltration in the basal septal inferior and posterior, LGE present in the lower basal and anterior basal septal. (**D**) Histological findings of fibrosis, mature adipocytes, and inflammatory cells. Courtesy from Cardiovascular Pathology, University of Padua.

Arrhythmogenic cardiomyopathy (ACM) is an inherited disease characterized by progressive myocyte loss with fibrofatty tissue replacement, which happens most often in the right ventricle (RV), but can affect the LV or both. In 30–60% of cases, the disease is familial, with the remaining cases having a de novo mutation, the PKP2 being the most frequently mutated gene. This mutation determines desmosome dysfunction, culminating in fibrosis. As part of the same disease spectrum, we also have Naxos disease and Carvajal syndrome [1]. Clinical findings of this disease can be seen in Figure 1.

In a registry from Veneto, Italy, ACM was the most common cause of SCD in athletes (24%) [21].

In an autopsy study of 118 athletes from the U.K. who had SCD, ACM was the second identified cause of SCD (14%) [22].

There is no single exam able to provide a conclusive diagnosis of ACM; thus, the diagnosis is made based on major and minor criteria defined by the Revised Task Force Criteria [23].

The arrhythmic risk stratification is based on criteria as sustained VT or VF, RV ejection fraction (RVEF), LV ejection fraction (LVEF), non-neurocardiogenic syncope, and non-sustained VT [24,25].

In the ACM context, intense and even moderate competitive or recreational sports should be avoided. Different from other conditions, in ACM, sports practice not only conveys arrhythmic risk to that specific moment but also accelerates disease progression. A study evaluating genotype-positive/phenotype-negative patients observed that athletes developed the clinical disease earlier (30 vs. 41 years of age) and were more likely to develop ventricular arrhythmias and heart failure [26,27].

## 7. Long QT Syndrome

Congenital long QT syndrome (LQTS) is an inherited cardiac channelopathy related to the prolongation of the QT interval, or even a normal baseline QT interval but an increased sensibility to the effect of QT-prolonging drugs. Different mutations can cause the syndrome. By now, more than 600 types of gene mutations are known, giving name to 17 different autosomal dominant LQTSs. Between them, there are two syndromes with extracardiac findings: Andersen–Tawil syndrome (LQTS-7) and Tymothy syndrome (LQTS-8). There are two autosomal recessive forms, the Jervel and Lange–Nielsen syndrome types 1 and 2, both associated with hearing loss. Despite being numerous and clinically heterogeneous, LQTS-1, LQTS-2, and LQTS-3 are responsible for more than 90% of cases, LQTS-1 being the most frequent [1].

The diagnosis is clinical, and one of the most widespread ways to conduct it is based on a validation score, the Schwartz score. LQTS is estimated to affect one in every 2000 individuals, and if left untreated, the SCD rate would be between 0.33–0.9% per year [1].

The triggering events are different according to the type of LQTS. Schwartz et al. demonstrated that exercise, especially swimming, was the trigger in 62% of cardiac events in LQTS-1, 13% in LQTS-2, and 13% in LQTS-3. Moreover, in LQTS-1, exercise was the trigger in 68% of arrhythmic events. On the other hand, for LQTS-2, loud and sudden auditory stimuli were the main trigger; and in LQTS-3, vagal situations such as sleep [28].

The 2015 ESC Guidelines on Prevention of SCD only restrict vigorous swimming in patients with LQTS-1. Other LQTS types or sports are not mentioned. The 2015 U.S. guidelines do not restrict competitive sports in patients adequately treated and asymptomatic for at least 3 months, with the exception of swimming for LQTS-1 individuals. The same guidelines do not restrict any activities in genotype-positive/phenotype-negative ones, given the proper preventive measures [1,29].

In a study cohort led by Johnson et al., 353 patients with LQTS clinically or only genotype positive were evaluated. After outpatient evaluation, treatment, and counseling, 130 patients (54% were genotype-positive/phenotype-negative) decided to keep competing in sports, 20 of those with an ICD implanted. During an average follow-up of 5.5 years, only one had a ventricular arrhythmia treated by an ICD shock [30].

## 8. Anomalous Origin of the Coronary Arteries

Anomalous origin of a coronary artery is a congenital disease with 0.17–1.3% prevalence in the general population. This disease has a wide range of presentations, i.e., it can be simply an independent origin of the anterior descending artery and the circumflex, or even a higher or lower origin in the same cusp. However, only the contralateral origin plays a role as an SCD risk factor. Even when it originates in the contralateral cusp, it may be a benign condition since it may take many routes to reach the contralateral side, but only the interarterial path (between the aorta and pulmonary trunk) has been shown to increase the risk of SCD. The proposed mechanism of SCD is ischemia triggered ventricular arrhythmia once exercise tends to induce expansion of aortic root and pulmonary trunk, and both may compress the passing coronary artery [31].

The clinical presentation, other than SCD as the first manifestation, can include angina, palpitations, syncope, and/or disproportionate dyspnea exercise triggered. It is remarkable that SCD risk tends to decrease with aging; this can be explained by aortic wall stiffening. A suspect case must be evaluated by direct angiography or by coronary tomography. In confirmed cases, presenting aborted SCD or induced ischemia, invasive treatment is indicated, which can be surgical or percutaneous [31].

These anomalies are the second cause of SCD in athletes in the USA and the third in Veneto, Italy [18,21]. The 2015 AHA guidelines restrict activities in patients with left coronary artery originating from the pulmonary trunk to only low-intensity sports; for those with left coronary originating from the right cusp, if interarterial, all competitive sports are prohibited, except class Ia (e.g., yoga, golf, bowling); for those with the right coronary originating from the left, with symptoms of ischemia in stress exams, all competitive sports are prohibited, except class Ia; if asymptomatic and negative exams, permission to compete should be granted after counseling. Patients after corrective cardiac surgery can return to competitive sports after 3 months and negative stress testing [2].

## 9. Myocarditis

Myocarditis is myocardial inflammation triggered by external antigens (viruses, bacteria, parasites, toxins, drugs, etc.) or autoimmune response. Viruses are the etiology most of the time, the most frequent being the parvovirus B19 [32].

A study using the International Classification of Diseases (9th revision) codes estimated the global prevalence of myocarditis to be approximately 22/100,000 patient-years [33].

The clinical manifestations of myocarditis are heterogeneous, ranging from virtually asymptomatic to severe myocardial destruction yielding cardiogenic shock and arrhythmias. The diagnosis is sometimes challenging, but cardiac magnetic resonance (CMR) has evolved and nowadays is the main tool for the 2018 Lake Louise criteria for myocarditis diagnosis [34].

In athletes’ autopsy studies, myocarditis was present in up to 8%. Despite appearing low in frequency, in some cohorts, it is the third cause of sports-related SCD. The risk of sudden death caused by myocarditis does not appear to correlate with the severity of myocardial inflammation [35].

The 2015 AHA/ACC Eligibility and Disqualification guidelines postulate that the athlete, after acute myocarditis, shall stop the activities and undergo new evaluation of serum markers, Holter, echocardiogram, and exercise ECG after 3 to 6 months. The athlete can resume training if the LV function is in normal range, serum markers are normal, and clinically relevant arrhythmias are absent on Holter. The routine use of CMR in this context is still debated [2].

## 10. Coronary Artery Disease

Atherosclerotic disease is the leading cause of SCD in athletes over 35 years old (>60%). It can also cause SCD in young individuals with severe metabolic disease or familial hyperlipemia. It can cause SCD in the sports context due to plaque rupture and consequent coronary occlusion leading to ischemia and ventricular arrhythmias or vasospasm [2].

In a cohort from Germany, 144 sports-related SCD were evaluated (mean age: 46.8 years old) in 66 patients. The cause of death was clearly defined, and from this group, 34 patients had myocardial infarction as the triggering event [36].

A prospective cohort study led by Marijon et al. evaluated 1247 sudden cardiac arrests, all patients between 35 and 65 years old (mean age 51.1 years old). Of these, 63 (5%) occurred during sports activities, and in 43 (68%), the etiology could be identified. Coronary artery disease was identified as the cause in 84% [37].

In patients with atherosclerotic coronary disease, two main subgroups need to be differentiated: those with a clinically manifested disease (previous infarction, angina, stress-induced ischemia) and those with a concealed disease (asymptomatic, without induced ischemia, disease identified by coronary tomography). This division comes from the fact that the risk is much lower for those with a concealed disease. For both groups, the exams necessary for decision making are the maximal stress ECG and any method to evaluate the LV function. For both groups, in those with normal LV function and no inducible ischemia, there is no restriction to exercise, but keeping the optimized pharmacological treatment is strongly advised. If the patient does not fulfill the above criteria, it is advisable to practice only low-intensity activities. In the context of post-infarction and/or post-revascularization, the minimum time of competitive sports restrictions is 3 months [2].

## 11. Ventricular Pre-Excitation

Wolff-Parkinson-White (WPW) accounts for approximately 1% of SCD in athletes, as demonstrated in a long-term registry. However, this is likely to be underestimated due to the lack of ECG screening [18]. Diagnosing WPW as a cause of SCD is difficult since autopsy examination cannot reliably identify the existence of accessory pathways. High-intensity physical activity increases the risk of WPW-related SCD, as observed in a study where two-thirds of the population suffered from an aborted VF cardiac arrest during exercise or under emotional stress [38]. In a recent Italian registry, exercise-related events were as high as 36% [39]. Even in sedentary individuals with pre-excitation, physical efforts of daily life may be an arrhythmia trigger. The physiopathology involves exercise-related adrenergic activation increasing vulnerability to re-entrant tachycardias and atrial fibrillation, which can precipitate ventricular fibrillation. The current recommendation on how to deal with athletes with ventricular pre-excitation states that athletes with short refractory period bypass tracts capable of anterograde conduction and a history of paroxysmal AF should have an ablation of the accessory pathway before clearance for competitive sports. In athletes with asymptomatic pre-excitation, it is reasonable to attempt risk stratification with stress testing to determine whether the pre-excitation abruptly terminates at low heart rates. If a low risk is unclear, it is reasonable to recommend invasive electrophysiological evaluation, with ablation of the bypass tract if it is deemed high risk for SCD because of a refractory period of 250 ms [2].

## 12. Commotio Cordis

Commotio cordis (CC) is an arrhythmic cause of sudden death of traumatic origin. It is caused by a blunt chest trauma leading to VF. It needs to be differentiated from cardiac contusion (contusio cordis), a situation in which the trauma causes structural cardiac damage, such as observed in motor vehicle accidents.

Most frequently, the affected individuals are young males, which can be explained by the overwhelming predominance of males in the sports in which commotio occurs. Among the sports, baseball is the one with the highest risk for CC. Nearly all CC events are caused by direct baseball strikes to the anterior chest. Other common sports in which CCs occur are hockey and softball, yet it has been described in nearly all sports. In those sports with no solid, hard ball, it can also occur due to impacts with elbows, fists, and helmets. A recently published registry gathered SCD data from soccer matches (2014 to 2018 period). Of the 617 SCDs reported, seven were confirmed to be due to commotio cordis, and another seven were very likely to be as well [40].

## 13. Sports in Patients with Implanted ICD

The concerns about sports practice in patients with ICD implanted are many. Between them is the fear about a possible failure of the shock to terminate a ventricular arrhythmia given the high adrenergic output intrinsic to vigorous exercise; the other is the possibility of bodily injury due to a syncopal event in the context of a competition; and last, the possibility of ICD system damage due to mechanical stress/trauma. All these concerns motivated the guideline authors to be restrictive in terms of sports practice in this context. Despite this, exercise restriction can have an impact on quality of life, mainly in children and adolescents that practice sports as a way to socialize.

The current recommendations provide the athlete with ICD implanted free participation in sports Ia (e.g., golf, bowling, yoga, etc.) as long as they are free of device therapy for 3 months. Participation in sports with higher peak static and dynamic components than class Ia may be considered if the athlete is free from ventricular arrhythmias requiring device therapy for more than 3 months. The decision regarding athletic participation should be made considering the likelihood of appropriate and inappropriate shocks and the potential for device-related trauma in high-impact sports [2].

## 14. Athlete’s Heart

It is critical that downstream testing, which can include cardiac imaging, exercise testing, and electrophysiological evaluation, is delivered and interpreted by physicians (typically cardiologists) who understand the cardiovascular adaptations to exercise training and resultant physiologic changes in the heart’s structure and function. This is the so-called athlete’s heart.

In most athletes, cardiac changes induced by exercise are modest and easily distinguishable from cardiac illnesses. However, in a small subset of athletes, vigorous training can be associated with more profound electrical and structural changes that may overlap with phenotypically mild manifestations of cardiac disease.

For example, sports with intense isometric exercise may induce left ventricular hypertrophy with wall thicknesses up to 12 to 14 mm in range, the same as that of mild HCM (gray zone hypertrophy) [41].

A small but significant proportion of endurance athletes will have dilated LV cavities with LV function in the inferior limit, which overlaps with findings of a dilated cardiomyopathy [42].

These physiologic changes in the LV may be accompanied by RV dilatation and reduced systolic function, which could raise concern for ACM in the appropriate context.

In these types of cases, it is crucial that an expert team evaluate the patient, including cardiologists familiar with the healthcare of athletes.

## 15. Clinical Work-Up to Prevent SCD in Athletes

### 15.1. Initial Evaluation

**Figure 2:**
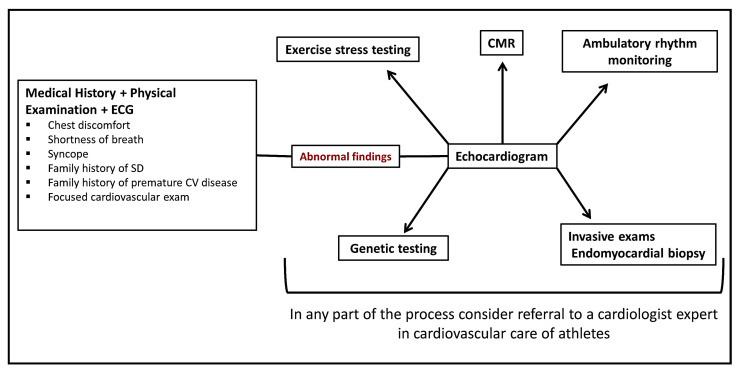
Suggested sports preparticipation evaluation work-up. ECG: electrocardiogram; SD: sudden death; CV: cardiovascular; CMR: cardiac magnetic resonance.

Since many of the cardiac conditions that cause SCD in athletes may not present warning symptoms, there has been considerable discussion about the role of preparticipation screening tests to evaluate for occult cardiovascular disease. A flowchart of screening and evaluating athletes is shown in Figure 2.

As demonstrated above, the incidence and causes of SCD vary widely depending on age, sex, race, country, and type of sport. The value of any screening test is determined by the characteristics of the population. Therefore, it is unlikely that a given single screening program will be effective across all groups.

The Sports Cardiology Study Group of the Working Group of Cardiac Rehabilitation and Exercise Physiology and the Working Group of Myocardial and Pericardial Diseases of the European Society of Cardiology recommend systematic preparticipation cardiovascular screening in young competitive athletes at the beginning of their activity, at the age of 12–14 years and at least every 2 years thereafter, to allow timely identification of progression of some diseases [43].

Accordingly, there are major differences in contemporary guidelines for the preparticipation screening of athletes. The American Heart Association (AHA) and American College of Cardiology (ACC) recommend screening that is limited to a targeted medical history and physical exam [44].

The specific 12-element list recommended by the AHA includes questions regarding a personal history of concerning cardiovascular symptoms (i.e., chest pain, syncope, dyspnea) and a family history of premature sudden death or disability from heart disease in addition to a focused physical exam. In contrast to the American recommendations, the European Society of Cardiology (ESC) and the International Olympic Committee (IOC) advocate for screening that also includes a resting 12-lead electrocardiogram (ECG) [45]. Thus, the role of the ECG in preparticipation screening has garnered considerable debate [46,47].

ECG-inclusive screening appears to increase the sensitivity of preparticipation screening for identifying cardiovascular disorders that predispose to SCD. Several guidelines and expert consensus statements have been designed to help clinicians interpret ECGs in athletes, with the goal of preserving sensitivity and improving specificity.

**Figure 3:**
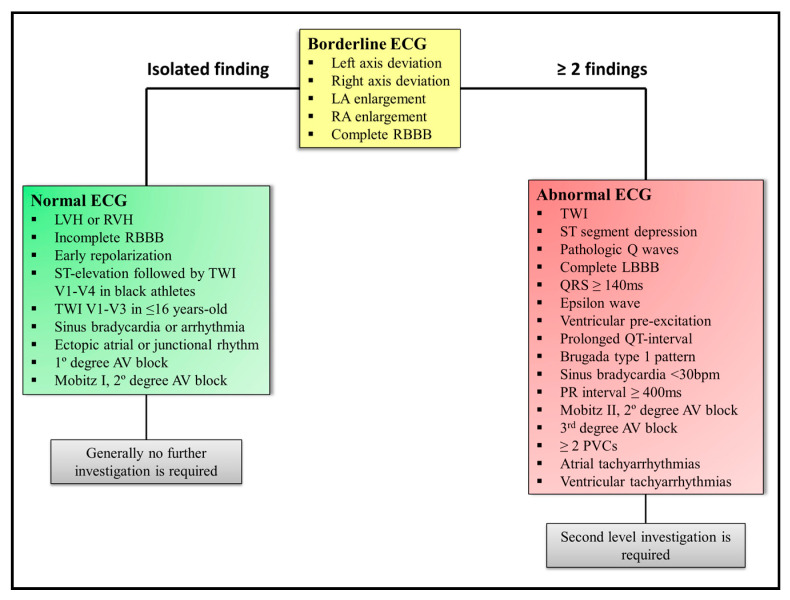
ECG interpretation flowchart according to the 2017 International Consensus. LA: left atrium; RA: right atrium; LVH: left ventricular hypertrophy; RVH: right ventricular hypertrophy; RBBB: right bundle branch block; TWI: T wave inversion; AV: atrioventricular; LBBB: left bundle branch block; PVC: premature ventricular beat.

The interpretation of ECGs in athletes is also complicated by the fact that exercise may be associated with a number of ECG findings that are not pathologic but can be perceived as such, especially by clinicians unaccustomed to interpreting ECGs in athletic individuals. Over time, the refinement in ECG criteria has led to a reduction in the false-positive rate. The last consensus added the borderline ECG concept and provided a more accurate evaluation [48]. The suggested criteria can be seen in detail in Figure 3.

This approach has important implications for the cardiovascular management of athletes, including preparticipation screening, clinical diagnosis, and risk stratification. An Italian study provided the most compelling evidence of the efficacy of ECG screening to save lives by identifying and disqualifying athletes with at-risk heart diseases. The annual incidence of SCD in athletes decreased by 89%, from 3.6 per 100,000 person-years in the prescreening period to 0.4 per 100,000 person-years in the late-screening period in the Veneto region [5].

Many of the causes of syncope can be suspected on the basis of ECG abnormalities at preparticipation screening [49].

### 15.2. Second and Third Level Examination

In cases of positive findings at any of the first-line examinations, the athlete should be evaluated with other invasive and/or noninvasive tests such as Holter, echocardiogram, exercise stress test, CMR, coronary computed tomography (CCT), invasive angiography, electrophysiological study with electroanatomical mapping, and endomyocardial biopsy (EMB) [49,50]. In certain contexts, the results of these exams may be mandatory to determine the eligibility of the subject for competitive sports.

Long-term ECG monitoring with Holter monitors can be useful in those patients with frequent or reproducible symptoms, such as palpitation or syncope. Athletes with intermittent, infrequent symptoms are best evaluated with a continuous loop monitor. These monitors continuously record a 1 to 3 min segment of surface ECG and, with activation by a button on the device, the tape is frozen, and the previous few minutes of the event are recorded.

Exercise testing should be adapted to the specific type of exercise/sport responsible for the arrhythmic events because a conventional exercise test may not replicate the specific clinical situation and the arrhythmogenic mechanism triggered by a given sport modality. An increase in the arrhythmia frequency at the beginning of exercise, disappearance at the peak of exercise, and reappearance during recovery usually suggests a benign behavior for premature ventricular beats. On the other hand, the triggering or worsening of ventricular arrhythmia with the increasing workload may point to an underlying cardiomyopathy or ion channel disease and may predict risk for malignant arrhythmias during sports [51].

In those athletes who are suspected of having an underlying cardiogenic substrate, a careful echocardiographic evaluation is usually the first choice as initial morphological evaluation. Despite the overall suitable diagnostic performance, transthoracic echocardiography (TTE) is typically normal in catecholaminergic ventricular tachycardia, anomalous coronary arteries, Brugada syndrome, and LQTS. TTE findings vary according to the cardiomyopathy. In ACM, they include RV and/or LV enlargement/hypokinesis, RV free wall thinning/dyskinesia, and prominent RV trabeculations, with a hyper-reflective moderator band. In HCM, we may find unexplained increased LV wall thickness (>13–15 mm). To differentiate from physiologic athlete’s hypertrophy, findings such as systolic anterior motion of the mitral valve, LV outflow tract gradient obstruction, and impaired diastolic function must be taken into account. LV remodeling in athletes is associated with normal or enhanced myocardial relaxation, leading to supranormal diastolic function, with findings as transmitral E/A ratio > 2, with typical low A wave velocity (late diastole). The tissue doppler imaging (TDI)-derived early diastolic myocardial velocity (e’) of the basal septal and basal lateral wall is increased, being responsible for a low E/e’ ratio. The RV has larger inflow and outflow dimensions in athletes compared to sedentary controls, with no impairment of the systolic function. Moreover, in highly trained endurance athletes, resting RV global systolic function is measured by fractional area change, and tricuspid annular plane systolic excursion (TAPSE) can be lower [52].

CMR has emerged as an important noninvasive, non-radiating imaging technique, particularly suited to provide detailed cardiac tissue characterization in athletes in the last decade [53]. Other than 3-D tomographic imaging with high spatial and temporal resolution, cine CMR imaging sequences allow clear delineation of the endocardial and epicardial borders, allowing precise wall thickness measurements in any LV segment [54]. CMR now plays an important role in the differential diagnosis of HCM versus athlete’s heart for several reasons [55]. First, CMR overcomes the difficulty of the TTE to obtain images in patients with a poor acoustic window. CMR has also proved to be of great importance in identifying the LV hypertrophy not seen on echocardiography, particularly when regions of increased wall thickness are completely (or predominantly) limited to focal areas of the LV wall such as the anterior free wall, posterior septum, and apex.

A recent study from Andreini et al. sought to determine whether CMR was capable of identifying structural heart disease (SHD) in patients with ventricular arrhythmia and normal echocardiography. A total of 946 patients were enrolled (mean 41 ± 16 years of age, 64% men). CMR detected SHD in 241 patients (25.5%) and abnormal findings not specific for a definite SHD diagnosis in 187 patients (19.7%). The most frequent cardiomyopathy detected in this cohort was myocarditis. All of them had a previous completely normal echocardiography [56].

Finally, myocardial fibrosis, identified in late gadolinium-enhanced images (LGE) taken 15 min post-contrast-enhanced CMR sequences can identify those patients who are at increased arrhythmic risk. In HCM, LGE is present in about half of the individuals. Myocarditis, in its turn, can be suggested by CMR in athletes through the identification of a number of abnormalities, including: (1) mildly or severely reduced systolic function, with or without regional wall motion abnormalities; (2) patchy or diffuse areas of LGE localized to the mid-myocardial or sub-epicardial layer with a non-coronary artery distribution; and (3) in the acute phase of the disease, areas of increased signal intensity on T2 weighted images due to edema [57].

CCT and coronary catheterization are also useful in the diagnosis of congenital anomalous coronary arteries and necessary in the diagnosis of atherosclerotic coronary disease.

### 15.3. Electroanatomical Mapping, Endomyocardial Biopsy, and Transcatheter Ablation

In those athletes who are suspected of having an underlying myocardial substrate for arrhythmias due to abnormal second-level examinations, performing an electrophysiological study and electroanatomical mapping can be useful.

The new imaging technologies are promising tools to better characterize the arrhythmic substrate. High-density electroanatomical mapping with the assessment of late, fragmented potentials and channels can help to localize the critical isthmus in complex arrhythmias. In addition, the real-time image integration of pre-acquired magnetic resonance and computed tomography information is feasible and accurate to assess epicardial fat and coronary artery, which are important issues during epicardial ablation [58].

The role of EMB in the diagnosis and treatment of adult and pediatric cardiovascular diseases remains controversial, and the practice varies widely even among cardiovascular centers of excellence. A need for EMB exists because specific myocardial disorders that have unique prognoses and treatment are seldom diagnosed by noninvasive testing [59].

In a recent study, Narducci et al. evaluated the role of an extensive diagnostic work-up in apparently healthy young patients with complex ventricular arrhythmias, comparing athletes and non-athletes. A total of 33 patients were enrolled: 18 competitive athletes (56%) and 15 non-athletes (44%). All of them underwent echocardiography and CMR that did not show structural disease. The diagnostic yield of EMB was 50% in athletes and 40% in non-athletes. Among athletes, the final diagnosis was myocarditis in 2, arrhythmogenic ventricular right cardiomyopathy in 1, and focal replacement fibrosis in 1 [60].

In athletes, the presentation of myocarditis is heterogeneous, and establishing the diagnosis is challenging with no current uniform clinical gold standard. The combined information from symptoms, electrocardiography, laboratory testing, echocardiography, cardiac magnetic resonance imaging, and in certain cases, endomyocardial biopsy can help to establish the diagnosis.

## 16. Conclusions

Sudden cardiac death in the athlete is a relatively rare event, but as sports practice is growing, and its occurrence is a tragedy, in absolute terms, it is a rather relevant issue. The causative disease is not the same through different population profiles. Therefore, the screening strategy must be adapted to the local demand. In general, a comprehensive clinical evaluation associated with ECG is enough for screening in most of the scenarios, and as a strategy, has been shown to reduce events in a classical cohort from Italy. In recent years, technological advances brought valuable tools that can be used in additional investigations such as the CMR and the endomyocardial biopsy.

## Figures and Tables

**Table 1:** Risk factors for sports-related sudden cardiac death.

**Young Athletes (≤35 yr)**
▪ Hypertrophic cardiomyopathy ▪ Arrhythmogenic right ventricular cardiomyopathy ▪ Anomalous origin of the coronary arteries ▪ Long QT syndrome ▪ Myocarditis ▪ Catecholaminergic polymorphic ventricular tachycardia
**Master Athletes (>35 yr)**
▪ Coronary artery disease ▪ Hypertrophic cardiomyopathy ▪ Arrhythmogenic right ventricular cardiomyopathy ▪ Myocarditis ▪ Long QT syndrome
**All ages**
▪ Male sex ▪ African-American ethnicity ▪ Vigorous exercise ▪ Exercise starter

## Data Availability

Not applicable.
